# A comprehensive resource of drought- and salinity- responsive ESTs for gene discovery and marker development in chickpea (*Cicer arietinum *L.)

**DOI:** 10.1186/1471-2164-10-523

**Published:** 2009-11-15

**Authors:** Rajeev K Varshney, Pavana J Hiremath, Pazhamala Lekha, Junichi Kashiwagi, Jayashree Balaji, Amit A Deokar, Vincent Vadez, Yongli Xiao, Ramamurthy Srinivasan, Pooran M Gaur, Kadambot HM Siddique, Christopher D Town, David A Hoisington

**Affiliations:** 1 International Crops Research Institute for the Semi-Arid Tropics (ICRISAT), Patancheru, Greater Hyderabad 502 324, AP, India; 2 Genomics Towards Gene Discovery Sub Programme, Generation Challenge Programme (GCP), c/o CIMMYT, Int. Apartado Postal 6-641, 06600, Mexico, D. F., Mexico; 3 Graduate School of Agriculture, Hokkaido University, Kita 9 Nishi 9, Kita-ku, Sapporo, 060-8589, Japan; 4 National Research Centre on Plant Biotechnology (NRCPB), IARI Campus, New Delhi-110012, India; 5 J. Craig Venter Institute (JCVI), 9704 Medical Center Drive, Rockville, MD 20850, USA; 6 Institute of Agriculture, The University of Western Australia (UWA) (M082), 35 Stirling Highway, Crawley WA 6009, Australia

## Abstract

**Background:**

Chickpea (*Cicer arietinum *L.), an important grain legume crop of the world is seriously challenged by terminal drought and salinity stresses. However, very limited number of molecular markers and candidate genes are available for undertaking molecular breeding in chickpea to tackle these stresses. This study reports generation and analysis of comprehensive resource of drought- and salinity-responsive expressed sequence tags (ESTs) and gene-based markers.

**Results:**

A total of 20,162 (18,435 high quality) drought- and salinity- responsive ESTs were generated from ten different root tissue cDNA libraries of chickpea. Sequence editing, clustering and assembly analysis resulted in 6,404 unigenes (1,590 contigs and 4,814 singletons). Functional annotation of unigenes based on BLASTX analysis showed that 46.3% (2,965) had significant similarity (≤1E-05) to sequences in the non-redundant UniProt database. BLASTN analysis of unique sequences with ESTs of four legume species (*Medicago*, *Lotus*, soybean and groundnut) and three model plant species (rice, *Arabidopsis *and poplar) provided insights on conserved genes across legumes as well as novel transcripts for chickpea. Of 2,965 (46.3%) significant unigenes, only 2,071 (32.3%) unigenes could be functionally categorised according to Gene Ontology (GO) descriptions. A total of 2,029 sequences containing 3,728 simple sequence repeats (SSRs) were identified and 177 new EST-SSR markers were developed. Experimental validation of a set of 77 SSR markers on 24 genotypes revealed 230 alleles with an average of 4.6 alleles per marker and average polymorphism information content (PIC) value of 0.43. Besides SSR markers, 21,405 high confidence single nucleotide polymorphisms (SNPs) in 742 contigs (with ≥ 5 ESTs) were also identified. Recognition sites for restriction enzymes were identified for 7,884 SNPs in 240 contigs. Hierarchical clustering of 105 selected contigs provided clues about stress- responsive candidate genes and their expression profile showed predominance in specific stress-challenged libraries.

**Conclusion:**

Generated set of chickpea ESTs serves as a resource of high quality transcripts for gene discovery and development of functional markers associated with abiotic stress tolerance that will be helpful to facilitate chickpea breeding. Mapping of gene-based markers in chickpea will also add more anchoring points to align genomes of chickpea and other legume species.

## Background

Chickpea is a member of the Leguminosae family, which includes 18,000 species, grouped into 650 genera [1] grown in semi-arid regions of the world. Chickpea, the world's third most important food legume is grown in over 40 countries representing eight geographically diverse agro-climatic conditions. In addition to being a major source of protein for human food in semi-arid tropical regions, chickpea crop plays an important role in the maintenance of soil fertility, particularly in the dry, rainfed areas [2,3]. The crop is a self-pollinated diploid (2x = 2n = 16 chromosomes) with a relatively small genome size of around 740 Mb [4]. Considering the small genome size, short seed-to-seed reproductive cycle of approximately three months and most importantly high economic importance as a food crop legume, chickpea is an interesting system for genomics research.

Majority of the world's chickpea is grown in South Asia and India being the largest producer with an estimated annual production of 5.9 million tonnes (mt). Total world production averages up to 9.3 mt [5], but there remains a gap between demand and supply due to the losses in the productivity caused by various abiotic and biotic stresses. Global annual production losses due to abiotic stresses alone are estimated to be around 3.7 mt, which amounts to 40-60% average loss.

Drought and salinity are two of the most important abiotic stresses that alter plant water status and severely limit plant growth and development. Drought causes a considerable (~50%) annual yield losses. Chickpea often suffers from terminal drought which delays flowering and affects yield. Plants adapt to drought stress either through escape, avoidance or tolerance mechanisms. Tolerating drought by developing deep root systems has been observed in chickpea [6]. Salinity is no less an important constraint for chickpea yield reduction. The continued depletion of ground water level and demand for irrigation has led to the salinization of arable lands. Hence, it is imperative to develop sustainable cultivars tolerant to drought and salinity. Factors such as high morphological and narrow genetic variation of the chickpea make it difficult to produce superior cultivars with durable resistance to the biotic and abiotic stresses through conventional breeding approaches. In this context, molecular markers or genes associated with resistance/tolerance to biotic/abiotic stresses should facilitate breeding practices by using marker-assisted selection [7]. In crop such as chickpea, where limited genomic resources are available, identification of stress-responsive genes can be undertaken by generating expressed sequence tags (ESTs) from stress-challenged tissues. EST sequencing projects have been contributing to gene discovery and marker development e.g. simple sequence repeats (SSRs) and single nucleotide polymorphisms (SNPs), as well as providing insights into the complexities of gene expression patterns and functions of transcripts in several crop species [8]. In the case of chickpea, however only a limited number of ESTs (7,097 ESTs at the time of analysis as of March 2008) are available in the public domain [9]. Very recently a set of 80,238 chickpea sequences of 26 bp have been added through SuperSAGE technique [10]. However, lack of availability of a chickpea reference genome limits the value of SuperSAGE tags, as only a fraction of them could be annotated.

In view of the above, the present study was undertaken to generate a comprehensive resource of drought- and salinity-responsive ESTs in chickpea with following specific objectives: (i) to generate drought-responsive ESTs from water-stressed root tissues of both drought-tolerant and drought-sensitive genotypes, (ii) to generate salinity-responsive ESTs from root tissues of NaCl treated plants of salinity-tolerant and salinity-sensitive genotypes, (iii) to identify unigenes of chickpea based on ESTs generated in this study as well as public domain ESTs, (iv) to functionally annotate the identified chickpea unigenes, (v) to identify correlated expression between genes, and (vi) to discover SSRs and SNPs for developing potential markers.

## Results

The relative effects of drought and salinity on the growth pattern were observed in all the objectives of the study. The growth of the drought-tolerant genotype (ICC 4958) was observed to be better compared to drought-sensitive genotype (ICC 1882) in all the cases of drought stress implications. Similarly, the salinity-sensitive genotype (ICCV 2) exhibited a relatively more stunted growth pattern than salinity-tolerant genotype (JG 11) when these genotypes were exposed to salinity stress. It was observed that the genotype JG 11 withstood salt stress (80 mM) to a greater extent in comparison to ICCV 2. However, when compared to the control set of plants in each case, growth of stressed plants was decreased. Root tissues from both drought and saline stressed plants were harvested for total RNA extraction and subsequent cDNA library construction.

### Generation of drought- and salinity-responsive ESTs

**Figure 1:**
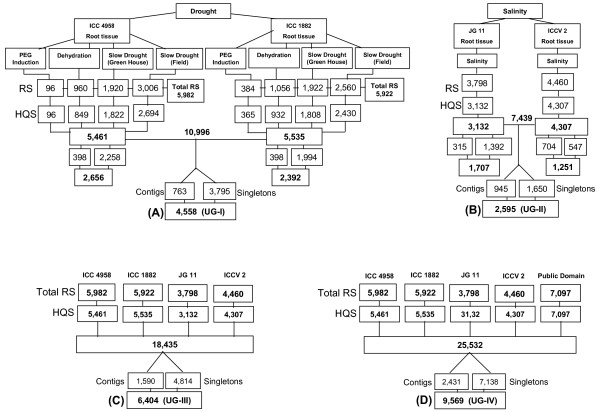
**Summary of ESTs generated from drought- and salinity-responsive chickpea genotypes**. The figure shows a flowchart of generation and analysis of ESTs in four groups. ESTs generated and analysed for drought-responsive tissues have been shown in **A**, for salinity-responsive tissues in **B**, all ESTs generated in this study in **C**, and all chickpea ESTs analysed in **D**. **A**: Four different drought stress treatments were imposed on each of chickpea genotypes ICC 4958 and ICC 1882. Raw sequences (RS) were trimmed to generate high quality ESTs (HQS). Cluster analysis of 10,996 sequences provided 4,558 unigenes (UG-I), **B**: ESTs were generated from salinity challenged root tissues of JG 11 and ICCV 2. Sequence trimming provided 7,439 high quality sequences. Clustering analysis of these sequences yielded 2,595 unigenes (UG-II), **C**: ESTs generated from four genotypes as shown in A and B were analysed together that provided a set of 6,404 unigenes (UG-III), **D**: ESTs generated in this study were analysed together with 7,097 public domain ESTs. Clustering and assembly analysis resulted in 9,569 unigenes (UG-IV) for chickpea.

A set of four genotypes i.e. ICC 4958 (drought-tolerant), ICC 1882 (drought-sensitive), JG 11 (salinity-tolerant) and ICCV 2 (salinity-sensitive) that represent parents of two mapping populations i.e. ICC 4958 × ICC 1882 and JG 11 × ICCV 2 segregating for tolerance to drought and salinity, respectively, were employed for generating ESTs. A total of 10 cDNA libraries including 8 from drought challenged tissues and 2 from salinity challenged tissues were generated. By using the Sanger sequencing approach, 5,982 and 5,922 ESTs were generated from ICC 4958 and ICC 1882 cDNA libraries. Similarly, 3,798 and 4,460 ESTs were generated from cDNA libraries derived from salinity stressed root tissues of JG 11 and ICCV 2, respectively. Details of EST generation from different cDNA libraries are given in Figure 1. In brief, a total of 20,162 ESTs were generated and after a stringent screening for shorter and poor quality sequences, 18,435 high quality ESTs were obtained. The average length of these high quality ESTs was 569 bp. All EST sequences were deposited in the dbEST division of GenBank (GR390696-GR410171 and GR420430-GR421115).

### EST assembly

**Figure 2:**
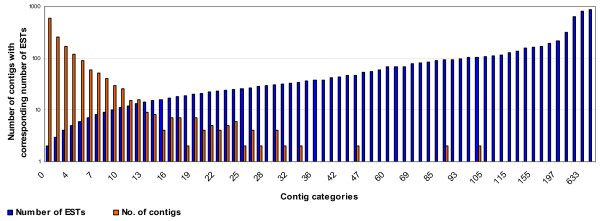
**Distribution of contigs according to the EST numbers**. Chickpea contigs were categorised based on the number of ESTs per contig. Blue bars indicate the EST size and the red bars indicate number of contigs belonging to respective EST size categories. Singletons were excluded from this analysis. Most of the contigs (88.5%) contain ≤10 ESTs, while 35 (2.2%) contigs comprise ≥29 ESTs and are represented once (contigs are not seen but corresponding ESTs can be seen in the graph).

Assembly analyses was done for different datasets of ESTs to define the unigenes for (a) drought-responsive ESTs, (b) salinity-responsive ESTs, (c) drought- and salinity-responsive ESTs, and (d) the entire set of chickpea ESTs including those from the public domain. These unigene (UG) sets are referred to UG-I, UG-II, UG-III and UG-IV, respectively. The UG-I comprised of 4,558 unigenes (763 contigs and 3,795 singletons) based on cluster analysis of 10,996 high quality drought-responsive ESTs. Likewise, the UG-II included 2,595 unigenes (945 contigs and 1,650 singletons) after cluster analysis of 7,439 high quality salinity-responsive ESTs. Based on the clustering of all the18, 435 high quality ESTs generated in this study, the UG-III was defined with 6,404 unigenes (1,590 contigs and 4,814 singletons). Detailed cluster analysis of the 18,435 ESTs identified 1,855 (10.06%) unique to ICC 4958, 1,606 (8.71%) to ICC 1882, 967 (5.24%) to JG 11 and 386 (2.09%) to ICCV 2. Inclusion of 7,097 ESTs available in the public domain at the time of analysis (as of March 2008), the entire set of chickpea ESTs (including 18,435 high quality ESTs generated in the present study and 7,097 available in public domain), the UG-IV was defined with 9,569 unigenes (2,431 contigs and 7,138 singletons). The assembly size in terms of number of ESTs aligned in each contig varied from 2 EST members (587 contigs) to 874 EST members (1 contig) with an average of 8.56 (Figure 2).

### Sequence annotation

Sequence annotation was performed for all four unigene datasets (i.e. UG-I, UG-II, UG-III and UG-IV) using standalone BLASTN and BLASTX algorithms. For BLASTN analysis, significant similarity was considered at threshold E-value of ≤1E-05. BLASTN similarity search for all the four unigene datasets was carried out against ESTs of closely related legume and model plant species. For instance, analysis of UG-III unigenes showed high similarity to *Medicago *(64.5%), followed by soybean (62.3%), *Lotus *(50.6%), poplar (42.8%), *Arabidopsis *(40.9%), groundnut (29.7%), and least to rice (27.0%). The BLASTN similarity results across different plant species for UG-III found 4,654 (72.6%) unigenes with significant similarity to ESTs of atleast one analysed legume species, 3,117 (48.6%) unigenes with significant similarity to ESTs of atleast one of the analysed model plant species and overall 4,719 (73.6%) unigenes with significant similarity to ESTs of atleast one of the analysed plant species. In contrast, 37 (0.5%) and 36 (0.5%) unigenes did not match ESTs of any legume or model plant species respectively. Results of the detailed analyses of the four unigene sets are given in Table 1.

**Table 1:** Analysis of chickpea unigenes with related legume and plant ESTs

	UG-I	UG-II	UG-III	UG-IV
High quality ESTs generated	10,996	7,439	18,435	25,532
Unigene assemblies	4,558	2,595	6,404	9,569
**Legume ESTs**				
Chickpea (7,097)	1,397	860	1,871	5,043
	(30.6%)	(33.1%)	(29.2%)	(52.7%)

*Medicago *(249,625)	2,668	2,216	4,131	6,568
	(58.8%)	(81.9%)	(64.5%)	(68.6%)

Soybean (880,561)	2,531	2,063	3,996	6,301
	(55.5%)	(79.6%)	(62.3%)	(65.8%)

*Lotus *(183,153)	2,023	1,734	3,244	5,102
	(44.3%)	(66.8%)	(50.6%)	(53.3%)

Groundnut (41,489)	1,245	1,064	1,908	2,978
	(27.3%)	(41.0%)	(29.7%)	(31.1%)

Significant similarity with ESTs of	3,055	2,314	4,654	7,815
atleast one legume species	(67.0%)	(89.1%)	(72.6%)	(81.6%)

Significant similarity across ESTs	678	160	284	552
of all legume species analysed	(14.8%)	(6.1%)	(4.4%)	(5.7%)

No similarity with legume ESTs	42	1	37	53
	(0.9%)	(0.03%)	(0.5%)	(0.5%)

**Model plant ESTs**				
*Arabidopsis *(1,527,298)	1,643	1,383	2,620	4,325
	(36%)	(53.2%)	(40.9%)	(45.1%)

Rice (1,240,613)	1,189	822	1,734	2,063
	(26.0%)	(31.6%)	(27.0%)	(21.5%)

Poplar (418,233)	1,723	1,437	2,744	4,391
	(37.8%)	(55.3%)	(42.8%)	(45.8%)

Significant similarity with ESTs of	1,945	1,597	3,117	5,088
atleast one model plant species	(42.6%)	(61.9%)	(48.6%)	(53.1%)

Significant similarity with ESTs of	1,020	760	1,521	1,742
all model plant species analysed	(22.3%)	(29.2%)	(23.7%)	(18.1%)

Significant similarity with ESTs of	3,114	2,319	4,719	7,881
atleast one plant species analysed	(68.3%)	(89.1%)	(73.6%)	(82.3%)

Significant similarity with ESTs of	480	131	228	283
all plant species analysed	(10.5%)	(5.0%)	(3.5%)	(2.9%)

No similarity with ESTs of any	40	1	36	38
plant species	(0.8%)	(0.03%)	(0.5%)	(0.3%)

**Figure 3:**
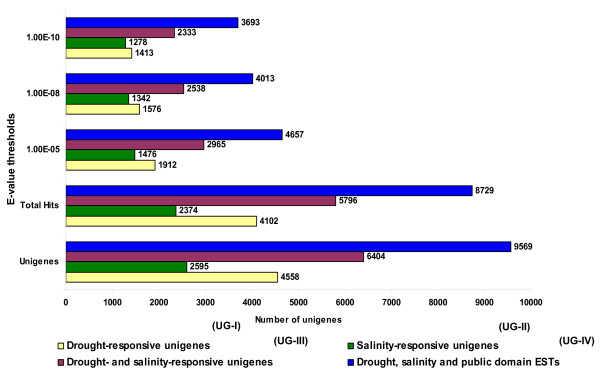
**Functional annotation of chickpea unigenes**. All four unigene groups (UG-I, UG-II, UG-III and UG-IV) were used for BLASTX analysis. Total hits (i.e. unigenes that showed similarity with the UniProt database sequences at E-value below or above IE-05) as well as significant hits at three different thresholds (≤1E-05, ≤1E-08 and ≤IE-10) for all the four unigene sets have been shown. Values against each bar represent number of unigenes showing significant annotations to sequences in the UniProt database at different E-value thresholds.

BLASTX search results for all four unigene sets against the UniProt database, found varying numbers of unigenes from different unigene sets with significant similarity at different thresholds. For UG-III (6,404), for instance, 2,965 unigenes had significant similarity against the UniProt database at E-value ≤1E-05, 2,538 unigenes at E-value ≤1E-08 and 2,333 unigenes at E-value ≤1E-10. Based on these findings, for further analyses of the BLASTX hits in this study, a threshold E-value ≤1E-05 was considered. Using this criterion, UG-I, UG-II, UG-III and UG-IV had significant similarity to 1,912 (41.94%), 1,476 (56.87%), 2,965 (46.29%), and 4,657 (48.66%) unigenes, respectively (Figure 3). Details of BLASTN and BLASTX analyses against closely related legume and model plant EST databases and the Uniprot database for all the four unigene sets are provided in Additional files 1, 2, 3 and 4.

### Functional categorization

Transcripts with significant BLASTX homology (≤1E-05) to annotated ESTs were further classified into functional categories. As expected only a small percentage of unigenes (~35.2%) could be thus classified. The Gene Ontology annotation of transcripts helped classify functional descriptions into three principal ontologies: molecular function, biological process and cellular component. Like in earlier studies of this nature [11], one gene product could be assigned to more than one multiple parental categories. Thus, the total number of GO mappings in each of the three ontologies exceeded the number of unigenes analysed. Details on GO analyses for all four unigene sets are provided in Additional files 5, 6, 7 and 8. As an example, GO analysis has been described below for one unigene set (UG-III).

**Figure 4:**
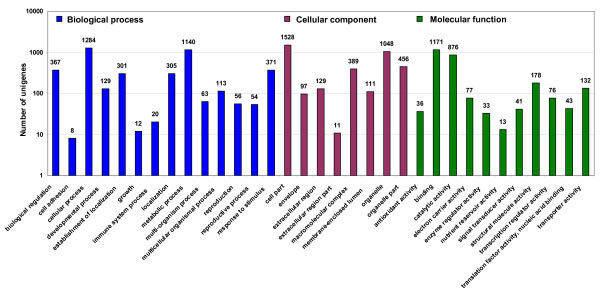
**Functional categorization of chickpea unigenes**. Functional categorization of 2,071 unigenes (UG-III) derived from ESTs generated in this study under three main categories: biological process, cellular component and molecular function.

The GO analysis of 2,965 (46.3%) unigenes from UG-III set (those with a significant hit in BLASTX analysis) revealed that 2,071 (32.3%) unigenes had GO descriptions for gene products: 1,684 were categorised under biological process, 1,586 under cellular component and 1,662 under molecular function. Of the functionally categorised unigenes, the largest proportion fell into cell part (1,528) followed by cellular process (1,284), nucleotide binding (1,171), metabolic process (1,140), organelle (1,048), catalytic activity (876) and response to stimulus (371) categories. Unigenes with significant similarity that could not be classified into any of the categories were grouped as 'unclassified'. Unigenes coding for housekeeping functions such as cellular process and metabolic process in the biological process ontology, cell part and organelle part in the cellular component ontology, and genes with binding and catalytic activity in molecular function category are over-represented in similar proportion in all unigene datasets (Figure 4). Enzyme Commission IDs were also retrieved from the UniProt database, to get an overview of the distribution of transcripts putatively annotated to be enzymes. The three largest groups of enzyme classes included transferases, hydrolases and oxidoreductases with 208 (27.9%), 206 (27.7%) and 183 (24.6%), respectively. The distribution pattern of enzymes was observed to be similar across all four unigene datasets.

### Correlated gene expression pattern analysis

**Figure 5:**
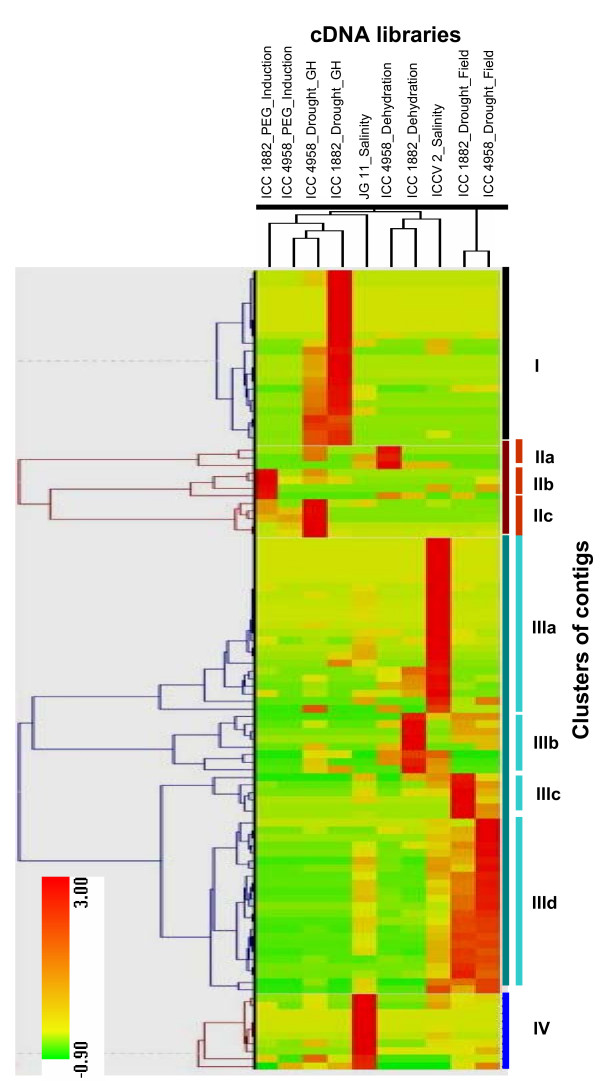
**Clustered correlation map of differentially expressed genes under stress**. Hierarchial clustering of ESTs representing genes involved in drought- and salinity- stress responses was done using HCE version 2.0 beta web tool. The dendrogram on top illustrates the relationship among 10 cDNA libraries: 1. ICC 4958_PEG_Induction, 2. ICC 4958_Dehydration, 3. ICC 4958_Drought_Glasshouse, 4. ICC 4958_Drought_Field, 5. ICC 1882_PEG_Induction, 6. ICC 1882_ PEG, 7. ICC 1882_Drought_Glasshouse, 8. ICC 1882_Drought_Field, 9. JG 11_Salinity, 10. ICCV 2_Salinity. Clustering of highly expressed ESTs (normalized using R statistics, R>8) into four major clusters (indicated by vertical colour bars) were further subclustered into nine groups based on library specificity (I, IIa, IIb, IIc, IIIa, IIIb, IIIc, IIId and IV). A colour map/heat map with red representing normalized expression values greater than the mean, green colour representing expression less than the mean and colour intensities in between representing the magnitude of the deviation from the mean can be observed. Colour scale (from green to red) represents the range of expression level.

To understand the patterns of gene expression and correlations between the 10 libraries from which ESTs were generated, the contigs generated in UG-III set were analyzed using the R Stekel statistical test [12] of IDEG.6 tool to identify the most significant expression and large differences in the abundances of ESTs in each contig. Of 1,590 total contigs in this dataset, only 105 returned a true positive significance (R>8) and were used for hierarchical clustering analysis. The expression level of each gene/contig (relative EST counts across all the libraries) has been graphically represented by a colour/heat map (Figure 5).

The expression profile of the 105 contigs with significant expression and their derivative libraries were classified into four major clusters (I-IV, represented in different colour bars) with the minimum similarity of 0.5 using HCE version 2.0 beta web tool. On the basis of their high expression level in a specific library, cluster II and III were further sub-clustered (IIa, IIb, IIc, IIIa IIIb, IIIc and IIId) that contained 3 (subcluster IIa) to 23 contigs (subcluster IIIa and IIId) representing different genes (Additional file 9). The cluster analysis showed higher number of differentially expressed genes in salinity libraries as compared to drought libraries. Furthermore as suggested by Mantri and colleagues [13], more transcripts were observed in severe stress-challenged libraries. In general, the cluster analysis revealed high expression of genes related to biotic stress signaling (20.9%), drought response (7.6%), transporter proteins (6.6%), reactive oxygen species (ROS) scavenging (4.7%) and transcriptional, translational regulation (6.6%) and uncharacterised proteins (7.6%) categories.

In addition, the clustering of different libraries was also analysed. The grouping/clustering of the 10 libraries was found consistent with their origin and genotypes. For instance, libraries were clustered into two main clades/clusters according to drought and salinity treatments. ICC 4958_Drought_Field and ICC 1882_Drought_Field libraries were grouped into the first clade, while the remaining libraries were grouped into second clade. The second clade was further divided into 2 clusters with both consisting of homogeneously segregating drought related libraries, while JG 11_Salinity and ICCV 2_Salinity cDNA libraries clustered heterogeneously within the hierarchical cluster. In both clades, libraries generated from similar conditions tended to cluster together, regardless of the genotype from which they derived, thus reflecting their relationship.

### Development of functional markers

In recent years, molecular markers have been developed from genes/ESTs and are popularly referred to as genic molecular markers (GMMs) [14] or functional markers [15] as a putative function can be deduced for majority of such markers. Functional markers (EST-SSRs and SNPs) were identified using unigene assembly UG-IV.

#### Identification of genic SSRs

EST-SSR markers can assay the functional genetic variation and also exhibit more transferability across taxonomic classes than genomic SSRs [16,17]. A total of 9,569 chickpea unigenes compiled in the present study (UG-IV) were analyzed using *MISA *(*MI*cro*SA*tellite) tool [18] for the identification of SSRs. As a result, a total of 3,728 SSRs were identified in 2,029 (21.2%) unigenes at the frequency of 1/707 bp in coding regions. Majority of SSRs, however, were monomeric repeats (1,793). Among other classes of SSRs, 126 dimeric SSRs, 110 trimeric SSRs, 7 tetrameric SSRs, 8 pentameric SSRs and 5 hexameric SSRs were also present (Table 2). Out of 3,728 SSRs, primer pairs were generated for 1,222 SSRs. After excluding the primers for monomeric repeats, a set of 177 primer pairs were considered. Considering minimum repeat number criteria such as six for di- and tri- nucleotides and four for tetra-, penta- and hexa-nucleotides, a sub-set of primer pairs were developed for only 77 SSRs.

**Table 2:** Features of SSRs identified in the chickpea unigenes

Total number of sequences examined	9,569
Total size of examined sequences (bp)	5,269,104
Total number of identified SSRs	3,728
Number of SSR containing sequences	2,029
Number of sequences containing more than one SSR	581
Number of SSRs present in compound formation	1,354
Frequency of SSR	1/700 bp
	
**Distribution of SSRs**	
Number of mononucleotide repeats	1,793
Number of di-nucleotide repeats	126
Number of tri-nucleotide repeats	110
Number of tetra-nucleotide repeats	7
Number of penta-nucleotide repeats	8
Number of hexa-nucleotide repeats	5

The potential of 77 SSR markers for detection of polymorphism was assessed on a set of 24 chickpea genotypes. Out of 77 primer pairs, 50 primer pairs yielded scorable amplicons. These SSR markers provided 1 (ICCeM0004, ICCeM0031, ICCeM0042, ICCeM0059 and ICCeM0073) to 12 (ICCeM0013, ICCeM0054 and ICCeM0055) alleles with an average of 4.6 alleles per marker. Only 45 primer pairs had more than one allele in the genotypes examined. The polymorphic markers showed a PIC value in the range of 0.08 to 0.86 with an average of 0.43 (Table 3).

**Table 3:** Diversity features of polymorphic EST-SSR markers

S. No	Marker name	SSR motif	Product size (bp)	**Allele No**.	PIC value
1	ICCeM0001	(TAA)_21_	224	6	0.58
2	ICCeM0004	(TC)_12_	116	1	0.00
3	ICCeM0005	(AAATGA)_5_	193	3	0.41
4	ICCeM0006	(TTC)_6_	212	4	0.53
5	ICCeM0007	(CT)_9_AA(CTT)_2_(TC)_4_TACT(CAA)_3_AG(A)_10_	207	5	0.60
6	ICCeM0011	(AG)_19_	215	7	0.76
7	ICCeM0012	(GATTC)_6_	107	2	0.30
8	ICCeM0013	(GAA)_6_(A)_14_	162	12	0.79
9	ICCeM0015	(AAT)_9_	182	4	0.24
10	ICCeM0017	(TCT)_9_	223	6	0.45
11	ICCeM0018	(GCTCCT)_5_	246	4	0.56
12	ICCeM0019	(GGAAA)_5_	207	3	0.54
13	ICCeM0025	(CT)_19_	260	6	0.62
14	ICCeM0027	(GAA)_10_	173	5	0.67
15	ICCeM0028	(TTTAT)_7_	275	2	0.25
16	ICCeM0029	(CAC)_6_(CTC)_6_	153	5	0.41
17	ICCeM0030	(A)_13_T(ATTT)_2_A(T)_14_	274	3	0.29
18	ICCeM0031	(ATC)_26_	204	1	0.00
19	ICCeM0032	(TA)_10_	215	6	0.60
20	ICCeM0033	(CT)_16_	162	10	0.86
21	ICCeM0034	(GA)_10_	124	2	0.09
22	ICCeM0035	(CT)_16_	181	8	0.74
23	ICCeM0036	(AC)_13_	275	4	0.38
24	ICCeM0037	(AG)_12_	171	2	0.15
25	ICCeM0038	(AACA)_6_	122	5	0.33
26	ICCeM0039	(GAAAGT)_5_	252	4	0.57
27	ICCeM0040	(TTC)_9_	254	4	0.46
28	ICCeM0041	(AAGTA)_6_	229	4	0.44
29	ICCeM0042	(TTAA)_5_	108	1	0.00
30	ICCeM0044	(AC)_37_	278	5	0.61
31	ICCeM0046	(TC)_9_	235	7	0.80
32	ICCeM0047	(CAT)_6_	210	3	0.31
33	ICCeM0048	(AG)_15_	134	7	0.57
34	ICCeM0050	(TC)_11_	198	5	0.63
35	ICCeM0051	(TC)_18_	275	8	0.79
36	ICCeM0053	(TTTTA)_5_	208	4	0.38
37	ICCeM0054	(TC)_22_	156	12	0.78
38	ICCeM0055	(TC)_8_TA(TC)_20_	177	12	0.85
39	ICCeM0056	(GTCATT)_5_	208	2	0.08
40	ICCeM0058	(AT)_16_	267	4	0.55
41	ICCeM0059	(TC)_14_	151	1	0.00
42	ICCeM0061	(TC)_13_	142	2	0.16
43	ICCeM0062	(TC)_12_	130	4	0.43
44	ICCeM0063	(TC)_12_	141	9	0.81
45	ICCeM0069	(AG)_19_	223	2	0.30
46	ICCeM0070	(CT)_21_	197	2	0.08
47	ICCeM0071	(AG)_17_	150	5	0.27
48	ICCeM0072	(TC)_17_	264	2	0.16
49	ICCeM0073	(GAG)_6_(GTG)_6_	244	1	0.00
50	ICCeM0076	(TC)_13_	241	4	0.37

#### Identification of SNPs

As large number of ESTs were generated from four genotypes, these EST datasets were analysed for identification of SNPs. SNP discovery was performed on contigs/multiple sequence alignments (MSA) containing two or more ESTs from more than one genotype. Out of 2,431 contigs (UG-IV), SNPs were detected in 2,047 contigs, while 384 did not have any SNP. A total of 36,086 SNPs were identified in 2,047 contigs. While 14,681 (40%) SNPs were identified in 1,305 contigs with 2-4 ESTs, the remaining 21,405 SNPs were identified in 742 contigs composed of 5 or more ESTs.

In order to perform cost-effective and robust genotyping assay for the 21,405 SNPs detected in 742 contigs, attempts were made to identify the restriction enzymes that can be used to assay SNPs via cleaved amplified polymorphic sequence (CAPS) assays. The analysis suggested that 7,884 SNPs could be assayed in 240 contigs by CAPS methods (Table 4).

**Table 4:** Identification of SNPs and CAPS based on the entire set of chickpea ESTs

Total number of contigs (UG-IV)	2,431
Total number of SNPs identified	36,086
Number of contigs containing SNPs	2,047
	
Contigs with 2-4 ESTs containing SNPs	1,305
Number of SNPs in contigs with 2-4 ESTs	14,681
Contigs with ≥ 5 ESTs containing SNPs	742
	
Number of SNPs in contigs with ≥ 5 ESTs	21,405
Number of contigs containing CAPS convertible SNPs	240
Number of SNPs which can be assayed by CAPS	7,884

## Discussion

A number of drought-responsive and salinity-responsive genes have been identified, cloned and characterized from an array of plant species and notably in model plant species such as *Medicago *[19], *Arabidopsis *[20], rice [21], soybean [22], *Lotus *[23] and poplar [24], etc. In contrast, in case of chickpea, where crop production is adversely affected by drought and salinity, not much information is available on candidate genes or molecular markers associated with tolerance/resistance to these stresses. This study attempted to develop a comprehensive transcriptomics resource of chickpea.

### cDNA libraries, ESTs and unigenes

Plant roots are the primary sites for perception and injury during water stress, including salinity and drought. In many circumstances, it is the stress sensitivity of the root that limits the productivity of the entire plant [25,26]. Plants are known to use more than one mechanism to resist unfavourable environmental conditions. For example, under drought conditions, plants may 'escape' the stress by undergoing rapid phenological development, completing their lifecycle before the onset of serious water deficit [27] or 'avoid' the stress by maintaining relatively high tissue water potential even at low soil-moisture content, balancing between water loss and turgor [28]. Therefore, root tissues were targeted for generating the drought and salinity-responsive ESTs in the present study.

With an objective of compiling as many drought-responsive and salinity-responsive ESTs as possible, different kinds of drought stress treatments (Polyethylene glycol (PEG) induction, dehydration, slow drought stress (dry down) in greenhouse and slow drought stress in field conditions) were imposed on drought-responsive genotypes, while salinity-responsive genotypes were stressed using 80 mM NaCl. This study provided 20,162 ESTs, 4,558 drought-responsive unigenes (UG-I), 2,595 salinity-responsive unigenes (UG-II), and a total of 9,569 chickpea unigenes (UG-IV). This is the first report on the generation of such large number of ESTs based on Sanger sequencing in chickpea. In the past, ESTs have been generated at ICRISAT [29] and by other research groups that represented a total of 7,097 ESTs in the public domain at the time of analysis in March 2008 [9]. Thus the present study contributes a 3 fold increase in ESTs.

### Characterization of chickpea unigenes

Four sets of unigenes were characterized in terms of their similarity with ESTs from other legume species available in the public domain and to deduce a putative function and assign them to a particular GO class. All the four unigene sets showed highest similarity with *Medicago *(58.8%-81.9%) and least similarity with groundnut (27.3%-41.0%). Other legume species showed similarity with chickpea unigenes in the range of 44.3% - 79.6% as shown in Table 1. The similarity of chickpea unigenes with other legume species is in general agreement with legume phylogenetic tree displaying relative order of speciation [30,31]. *Medicago *is the most closely related species in the phylogenetic tree which explains the larger number of significant hits with *Medicago *sequences. There were some exceptions however; chickpea unigenes showed higher similarity to soybean (65.8%) as compared to *Lotus *(53.3%) whereas the phylogenetic distance between chickpea and *Lotus *is less than it is with soybean. The smaller EST dataset available in *Lotus *(183,153) as compared to soybean (880,561) is probably responsible for this observation.

While analysing sequence similarity of compiled chickpea unigenes (UG-IV) with other legume species, 7,815 (81.6%) unigenes had significant similarity to ESTs of at least one analysed legume species. Conservation of 552 (5.7%) unigenes across the legume species was observed. Sequence comparison of UG-IV unigenes with other plant species i.e. rice, poplar and *Arabidopsis *showed 5,088 (53.1%) unigenes sharing significant similarity with ESTs of atleast one of the model plant species and 1,742 (18.2%) conserved across all three model plant species, while 283 (2.9%) of total chickpea unigenes shared significant similarity with ESTs of all the plant species analysed. This suggests that 283 (2.9%) chickpea unigenes can be considered conserved in plants; 552 (5.7%) unigenes can be considered conserved across legumes. It is also interesting to note that 53 (0.5%) chickpea unigenes did not show any homology with any of the legume EST datasets searched. This indicates that at least 53 (0.5%) new unigenes without legume homologs in the public domain have been contributed to through this study. About 38 (0.3%) chickpea unigenes did not show any similarity with sequences from any of the plant species EST datasets searched or UniProt database and may thus be considered novel.

In terms of BLASTX analysis, a putative function could be deduced for 2,965 (UG-III) of 6,404 unigenes and 2,071 could be functionally categorised based on ESTs generated in this study. Analysis of the entire chickpea unigene set (UG-IV) revealed that only 3,147 (32.8%) out of 9,569 unigenes could be functionally categorised. The functional annotation will be very useful in selecting unigenes for developing microarray or functional molecular markers [32,33].

The functional categorization of each unigene dataset into GO categories revealed a similar percentage distribution of genes in the four categories: cellular process, metabolic process, binding and catalytic activity. These four functional categories described were also reported as major categories in *Arabidopsis *[20], rice [34], tomato [35] and also in barley [33].

### Clustering analysis to identify patterns of gene expression

Clustering analysis identified patterns of gene expression which are unique to different libraries. The profiles of some of the interesting gene families and genes that could play an important role in stress response were investigated (Figure 5).

The majority of the genes in cluster I (containing 22 contigs) are those highly expressed in ICC 1882_Drought_GH library. Apart from very highly represented 'Isoflavone-7-*O*-methyltransferase 9' (7 IOMT-9) (UniProt ID: O22309, 93 transcripts), an isoflavanoid biosynthetic enzyme which plays a role in plant resistance to pathogens [36], many transcripts with putative annotations to nucleic acid binding, protein binding activity classes were co-expressed in this cluster. Two un-annotated contigs (Contig640 and Contig720) identified in this cluster were considerably expressed in ICC 1882_Drought_GH library with transcript numbers of 150 and 449, respectively. Considering the expression ratio difference of these uncharacterised proteins, further studies may reveal their possible role during stress conditions in plants.

The sub-cluster IIa contains genes specifically expressed in the ICC 4958_dehydration root library. Two contigs homologous to the biotic stress-responsive 'probable pleiotropic drug resistance' (*PDR*) protein (UniProt ID: Q7PC86) and 'phenylalanine ammonia-lyase' (*PAL*) (UniProt ID: P45732) were identified. Both *PDR *and *PAL *are involved in the phenylpropanoid and flavanoid/isoflavanoid pathways leading to phenylpropanoid biosynthesis in plants in response to various biotic and abiotic stresses [37,38]. The relative transcript counts of *PDR *and *PAL *signify their associated role in stress signaling processes.

The gene coding for 'dead ringer protein homolog' (UniProt ID: Q8MQH7) was found to be highly induced in ICC 1882_PEG_Induction library of subcluster IIb. Functionally, these proteins protect other proteins from denaturation by heat [39]. Their up-regulation in the drought tolerant genotype suggests a significant role in imparting tolerance against drought.

All 23 contigs in the sub-cluster IIIa were highly expressed in the ICCV2_Salinity library. UniProt classification of these contigs indicated that a large fraction of them (11) were involved in cellular processes and nine contigs homologous to different stress related proteins. 'ABA responsive' (ABR) related transcripts were observed higher in drought- and salinity-sensitive genotype specific libraries such as ICC 1882_PEG and JG 11_Salinity libraries. Higher accumulation of ABR transcript levels has been reported in stressed plants [40]. Similarly, high accumulations of 'heat shock cognate 70 kDa protein' (HSP70s) (UniProt ID: P27322) was observed in stress-sensitive genotype related ICCV2_Salinity root library as compared to JG 11_Salinity and ICC 1882_Drought_Glasshouse libraries. This implies a significant role of 'HSPs' in protecting the plant cells during abiotic stresses. Interestingly, the co-expression of Contig773, Contig1406 and Contig1457 corresponding to 'NAD(P)H-dependent 6-deoxychalcone synthase' (UniProt ID; P26690), 'Isoflavone-7-O-methyltransferase 9' (UniProt ID; O22309) and 'Isoflavone reductase' (UniProt ID; Q00016), respectively were observed in the subcluster IIIa. All these genes were involved in the phytoalexin biosynthesis pathway and isoflavanoid phytoalexin has been reported to occur principally in legumes during defence response of plants [41].

The sub-cluster IIIb represents genes that are highly expressed in the ICC 1882_dehydration library, accounting for 7.6% of all contigs in the hierarchial cluster. Six out of eight genes co-expressed in this cluster have been putatively annotated and categorized to stress response related genes. Contigs coding for 'dehydrin' DHN3 (Contig91) (UniProt ID: P28461) and hydrophilic 'late embryogenesis abundant (LEA) protein 1' (UniProt ID: Q49816; Q49817) (Contig638 and 534) were co-expressed in this cluster. Involvement of LEA-1 genes has been reported during seed development at low water potentials [42]. The presence of protective compound such as 'sugar transport protein 13' (UniProt ID: Q94AZ2) during water stress conditions [43] in this cluster suggests a protective reaction to osmotic stress in sensitive genotypes as compared with tolerant genotypes. Similarly, the occurrence of 'glycine-rich cell wall proteins' (GRPs) (UniProt ID: A3C5A7) involved in cell wall lignification during pathogen attack [13,44], 'pathogenesis-related protein 1A/1B precursor' (UniProt ID: P32937) and 'chitinase-3-like protein 4 precursor' (UniProt ID: Q91Z98) in this cluster signifies their protective role during stress response. These observations suggest the possible involvement of the above mentioned genes in protection and repair of damaged cell walls caused under stress.

The sub-cluster IIId includes 23 (21.9%) contigs whose transcripts are highly expressed in both the ICC 4958_Drought_Field library and ICC 1882_Drought_Field library and also contains a majority of stress-responsive functionally important genes. Up-regulation of membrane spanning genes such as 'probable aquaporin *PIP*-type' (UniProt ID: P25794; Q9ATM4), which mediate regulation of root hydraulic conductivity in response to environmental stimuli [45] were observed also in ICCV2_Salinity library as well as their significant expression in the other two libraries mentioned above and is not unexpected since its involvement is well reported in plant drought stress response. It is noteworthy that the transcripts annotating to 'metallothionein- like proteins' (MTs) (UniProt ID: Q39458, Q39459, Q9SSK5) involved in heavy metal detoxification and accumulate in response to high metal concentration, nutrient deprivation and heat shock [46], identified in this cluster were highly expressed in ICC 4958_Drought_Field library and even more so in ICCV 2_Salinity library. Their high expression level in the ICCV 2_Salinity library may be correlated with their putative role in detoxification of salts accumulated in salinity stressed roots. Contigs similar to classical 'arabinogalactan proteins' (AGPs) (UniProt ID: Q9ZT16), abundant in the plant cell wall and plasma membrane [47] were identified to be highly expressed in the ICC 4958_Drought_Field library. 'Fasciclin like Arabinogalactans' (FLAs) is a class of AGPs known to be regulated both during developmental processes and stress responses. Different classes of FLAs have been associated with ABA and down-regulated by drought stress [48]. Likewise, a high copy number of transcripts corresponding to the ABR gene (UniProt ID: Q06931) were highly expressed in ICC 1882_Drought_Field library (423 transcripts), ICC 4958_Drought_Field library (291 transcripts) and moderately expressed in ICCV 2_Salinity library (55 transcripts). The predicted expression pattern of AGPs and ABRs identified in this study is also consistent with experimental observations in *Arabidopsis *[47].

It is important to note that although a high number of transcripts annotated to genes known to be involved in stress responses have been identified in clusters IIb, IIc, IIIc and IV, their functional inter-relativity could not be explained. In addition, many uncharacterised and unknown proteins with significant expression levels have also been identified in these clusters. Further investigation of these genes is required to understand their importance in stress signaling and/or tolerance mechanisms in chickpea. Although these genes have been identified in response to abiotic stress, many of these genes are likely to be involved in conferring resistance to biotic stresses as well, since a crosstalk between signal transduction occurring during biotic and abiotic stress is well known phenomenon [13]. In summary, these candidate genes will be very useful to integrate in genetic maps and link them with QTLs for abiotic/biotic stress tolerance as well as targeting them in genetic engineering or reverse genetics approaches.

### Transcriptome resource for developing functional markers

The chickpea unigene dataset (9,569) (UG-IV) defined in the present study can be used to develop a variety of molecular markers as stated by Varshney and colleagues [14]. In case the polymorphism detected by a particular type of molecular marker correlates with variation in coding region affecting gene function, the molecular marker could become a candidate marker for a trait of interest.

Mining of 9,569 unigenes (UG-IV) showed occurrence of 3,728 SSRs, however majority of these SSRs represent mononucleotide repeats (1,793). This is a common feature of database mining of ESTs for identification of SSRs [16,49]. After excluding the monomeric SSRs, higher proportion of SSRs was dominated by dimeric SSRs (126) followed by trimeric SSRs (110). In general, studies dealing with mining of ESTs for SSRs reported higher abundance of trimeric SSRs [16,50], however there are several studies [51] that show exceptions as well. Indeed the number and distribution of SSRs of a particular class also depends on the criteria and tool used for mining the ESTs [16].

In terms of converting identified SSRs into potential SSR markers for chickpea genetics and breeding, primer pairs could be designed for 177 SSRs (Additional file 10). This increases the repertoire of SSR markers available in chickpea [14]. The EST-SSR markers developed in this study were compared with those developed earlier and available in public domain [e.g.[52-55]] so that only non-redundant set of SSR markers should be developed. As a result, for validation purpose, only 77 SSR primer pairs with preferred criteria were synthesized. Fifty of these newly developed SSR markers on 24 chickpea lines, had varied alleles (average 4.6 per marker) per locus with an average PIC value of 0.43 per marker. This suggests a moderate discriminatory power of this new set of SSR markers. Di-nucleotide SSRs (ICCeM0011, ICCeM0046, ICCeM0048, ICCeM0035, ICCeM0051, ICCeM0063 and ICCeM0033) with atleast 12 repeat units and compound motifs (ICCeM0013, ICCeM0054 and ICCeM0055) had relatively higher number of alleles (7-12) and high PIC values ranging from 0.73 to 0.86. Such correlation between number of alleles, maximum number of repeat units and PIC value has been observed in other earlier studies [56].

The present study also provided 36,086 SNPs in 2,047 unigenes that can be used for converting into SNP markers. SNPs are the most abundantly found co-dominant polymorphic sites in greater proportion both in intronic and exonic regions of the genomes, occurring with variable frequencies and becoming very popular in plant genetics and breeding due to their amenability for high throughput genotyping. EST mining has been a popular approach for large scale identification of SNPs [57]. However, the error rates in EST sequencing may sometime lead to erroneous SNP calls. Therefore, to enhance more reliability of SNPs identified, deep multiple alignments (=5 reads per contig containing SNP) were considered. In case of chickpea, although a few SNP markers have been reported [58,59], the present study, probably provides the first comprehensive set of SNPs for chickpea. Although high-throughput SNP genotyping platform such as GoldenGate assay of Illumina are available that allows genotyping of large number of SNPs (e.g. 1,536) in parallel [60], conversion of SNPs into CAPS assay is a cost effective method that can be used in low-tech laboratories [61]. The present study provides 240 candidate genes where SNPs can be assayed by CAPS.

## Conclusion

In summary, the present study provides 20,162 new chickpea ESTs (6,404 unigenes) including 11,904 ESTs (4,558 unigenes) from drought challenged libraries and 8,258 ESTs (2,595 unigenes) from salinity challenged libraries. Including the 7,097 public domain chickpea ESTs in the analysis defined chickpea unigenes to 9,569. Five hundred and fifty two (5.7%) chickpea unigenes were conserved across legume species, 283 (2.9%) conserved across analysed plant species and 38 (0.39%) unigenes were specific to chickpea. This study provides an overview of expression patterns of 105 contigs/genes that were up- or down-regulated in response to imposed abiotic stresses, validated either by over expression or TILLING (Targeting Induced Local Lesions IN Genomes). These genes together with 177 SSR markers and 742 genes with SNPs provide a comprehensive resource for integration and development of the transcript map of chickpea. The EST resource generated in this study will significantly impact chickpea genetics, and breeding in general and for improving the crop for drought and salinity tolerance in particular.

## Methods

### Drought stress treatments

Two chickpea genotypes, ICC 4958 (drought tolerant) and ICC 1882 (drought sensitive), parents of a mapping population segregating for drought tolerance were selected. Four drought stress treatments were imposed to target wide expression profiles associated with drought: (i) PEG induction, (ii) dehydration, (iii) slow drought (dry down) under greenhouse conditions and (iv) slow drought under field conditions, to generate four libraries for each drought-responsive genotype.

In order to capture and study various drought-responsive ESTs, the two chickpea genotypes, ICC 4958 and ICC 1882 were first subjected to chemically induced dehydration stress using PEG. Various concentration of PEG such as 50 mM, 10 mM, 5 mM and 1 mM were evaluated for optimizing a slow drought stress that would mimic the field drought condition in these two chickpea genotypes grown in hydroponic solution under greenhouse condition (data not shown). PEG concentrations of 50 mM and 10 mM were lethal. The relative water contents (RWC) in roots of ICC 4958 and ICC 1882 at different time intervals in other two treatments suggested a slower drought stress effect at 1 mM concentration than at 5 mM. Finally, a slow drought stress was imposed using 1 mM PEG and the intensity of the drought stress was assessed by recording the transpiration ratio (TR) on a daily basis. Root samples from the PEG-stressed plants of the two genotypes were harvested when the transpiration ratio reached 0.1. Here, in this study we recognized a relatively rapid stress imposition taking place with PEG treatment which hardly reflects the kinetics of stress imposition of the natural environment. Simultaneously, dehydration stress was imposed on another set of plants grown under similar growth conditions by removing the hydroponic solution from their trays and root samples were harvested when the relative water content (RWC) of these plant were observed between 50-60%. With another set of pot grown plants, a dry down experiment was conducted under greenhouse conditions as described by Ray and Sinclair [62] with 10 treatment and 10 control plants per genotype. Pots were allowed to dry through transpirational water loss until the TR reached 0.1. At this stage root samples were harvested from stress plants of each of the two genotypes. The slow drought stress under field conditions was conducted in a rain out shelter by sowing 10 seeds of the two chickpea genotypes in 8 inch pots containing a mixture of soil and sand (1:1). Drought stress was imposed when the plants reached 20-22 days old seedling stage. The root samples from the stressed plants were harvested when the TR reached 0.1 and were stored in -80°C for RNA extraction.

### Salinity stress treatment

The effect of salinity was studied in JG 11 (saline tolerant) and ICCV 2 (saline sensitive) chickpea genotypes, which are the parents of a mapping population segregating for salinity. Plants of both salinity-responsive genotypes were grown in pots (5 replicates) in greenhouse and treated with 80 mM NaCl solution at flowering stage. After a stress period of 5 days, root tissues from stressed plants of both genotypes were harvested for total RNA extraction and cDNA library construction.

### Chickpea cDNA libraries and EST generation

Total RNA was extracted from root samples representing libraries of different genotypes challenged with a range of drought and salinity stresses, using a modified hot-acid phenol method [63] followed by lithium chloride precipitation. The integrity and quantity of total RNA was assessed spectrophotometrically and also by formaldehyde agarose gel electrophoresis. cDNA was synthesized using Super SMART™ PCR cDNA Synthesis Kit (Clontech^®^, USA), according to the manufacturer's instructions. The purified cDNA was ligated into pGEM^® ^Easy vector (Promega^®^, USA) using T4 DNA ligase. Subsequently the cDNA-ligated vector was transformed by electroporation technique, applying 260 volts for 10 milliseconds into One Shot^® ^Top 10 Electrocomp™ cells (Invitrogen, USA). The transformed cells were incubated in LB medium at 37°C for 1 hour at 220 rpm. Subsequently, the transformed cells were plated on agar plates containing ampicillin (100 μg/mL), 40 ul X-gal (20 mg/mL) and 5 ul IPTG (200 mg/mL) and incubated at 37°C for overnight. Individual white colonies were randomly picked and transferred into 96-well plates (Nunc™, Denmark) and incubated at 37°C for 24 h while shaken at 220 rpm. Plasmid DNA was isolated from overnight grown cultures using an alkaline lysis method [64]. The concentration and quality of the plasmid DNA was assessed on 1.2% agarose gel and single pass Sanger sequencing (Macrogen Inc., Korea and J. Craig Venter Institute) was performed using universal M13 primer.

### Sequence processing

The sequence data files' containing raw sequence reads were subjected to two phases of screening. Primarily all the sequences were subjected to Sequencher™ 4.0 (Gene Codes Corporation, USA) to extract high quality regions from the remaining adjoining potential vector regions in the raw sequence data. A Perl script 'EST trimmer' [65] was used to eliminate poly-A tail and low quality sequences which had less than 100 bp. The CAP3 assembly program [66] was used to perform subsequent steps of clustering, sequence assembly, alignment analysis and consensus partitioning to derive contigs and singletons. This was done to mask the redundancy in the above libraries. In order to assess the transcript redundancy among the generated libraries and for downstream analyses, four different unigenes datasets were generated (i) drought-responsive ESTs from both ICC 4958 and ICC 1882 genotypes (UG-I); (ii) salinity-responsive ESTs from JG 11 and ICCV 2 genotypes (UG-II), (iii) total ESTs derived from both drought and salinity response generated in this study (UG-III), and (iv) all chickpea ESTs generated including 7,097 public domain ESTs (UG-IV).

### Sequence annotation

Standalone BLAST was used to obtain best matches for all unigene sequences derived after the CAP3 assembly program with a threshold E-value of ≤1E-05. BLASTN was performed against formatted sequence databases of legume species, such as *Medicago*, soybean, *Lotus *and groundnut, and also *Arabidopsis*, rice and poplar ESTs downloaded from NCBI [9]. BLASTX was performed against the UniProt non-redundant protein database.

### Functional categorization of annotated sequences

Functional assignment of unigenes was performed for all sequences finding significant hits in the UniProt database (≤1E-05). The Gene Ontology IDs were retrieved from the UniProt database using keywords obtained in the BLASTX descriptions of the most significant hits. Based on the Gene Ontology ID, unique sequences were categorised into three principal categories: biological processes, cellular localizations and molecular functions. Enzyme commission numbers were also extracted for corresponding unigenes and assigned to specific biochemical pathways.

### Correlated gene expression analysis

Gene expression pattern and correlation of genes expressed in response to abiotic stress regimes in the study were analyzed. Of 1,590 contigs (UG-III) 563 which had at least five ESTs from all the 10 libraries were extracted for expression profiling based on the EST counts for each library. The data matrix was subjected to R statistics (R>8) for identification of the most differentially expressed genes by using the web tool IDEG6 [67,68]. As a result, of the 563 normalized contigs only 105 contigs showed differential expression and were subsequently subjected to hierarchical clustering using HCE version 2.0 beta web tool [69].

### Identification of EST-SSRs

For identification of SSRs in ESTs, *MI*cro*SA*tellitepearl script [70] was used. *MISA *search provides information about the type and localization of each individual microsatellite and parses the calculated primer sequences, their sequence and melting point, melting temperature, and expected PCR product size.

### EST-SSR screening and data analysis

For assessing the potential of the newly developed EST-SSRs, the markers were screened on 24 different chickpea accessions which were obtained from the International Crops Research Institute for the Semi-Arid Tropics (ICRISAT), Patancheru, India (Additional file 11). Seventy seven newly synthesized M13 tailed EST-SSR primer pairs were chosen for the study. Polymerase chain reaction (PCR) was performed in 5 μL of a mixture containing 5 ng DNA, 2 pM of each primer, 2 mM dNTPs, 10 mM MgCl_2_, and 0.1 U of *Taq *DNA polymerase in 1× reaction buffer along with 2 pM dye to enable detection of the fragments in the ABI-3700 automated sequencing system. The steps of the PCR process are: (i) an initial denaturation step for 3 min at 94°, (ii) 5 cycles of 20 sec at 94°, 20 sec at 60° and 30 sec at 72° (iii) 40 cycles of 94° for 20 sec, 56°C for 20 sec and 72°C for 30 sec, and (iv) a final extension step for 20 min at 72°C. Data was analysed using GeneMapper^® ^Software v4.0. Allelic data obtained from GeneMapper analysis was submitted to Allelobin, an in-house programme that automates the process of assigning allele sizes to appropriate allele bins [71]. PIC value and other marker informations were obtained using PowerMarker v3.25 [72].

### Identification of SNPs

The SNP diversity estimator, 'Divest' [73], an in house Perl module was used to detect putative SNPs from the EST sequences. The ESTs reported in this study are from ICC 4958, ICC 1882, JG 11, ICCV 2, while the public domain ESTs came from Castellana, ICC 4958, Pusa 32, Pusa Pragathi and XJ-209 genotypes. The program uses CAP3 alignment output files as input to detect SNPs based on the base redundancy in sequence alignments. SNPs thus identified were converted to CAPS markers computationally using SNP2CAPS [74].

## Authors' contributions

RKV planned and coordinated the study and wrote the MS together with PJH and finalized the MS, PJH executed majority of experiments and helped RKV in writing and finalizing the MS, PL, JK, AAD and VV were involved in setting up drought and salinity experiments and isolation of RNA, JB was involved in bioinformatics analysis, YX and CDT sequenced several cDNA libraries, RS, PMG, KHMS and DAH collaborated with RKV in planning the study. All authors read and approved the final manuscript.

## Supplementary Material

Additional file 1 **UG-I BLAST analyses results**. Table showing BLASTN and BLASTX results of UG-I dataset with corresponding details of GB ID numbers, descriptions and E-value. Click here for file

Additional file 2 **UG-II BLAST analyses results**. Table showing BLASTN and BLASTX results of UG-II dataset with corresponding details of GB ID numbers, descriptions and E-value. Click here for file

Additional file 3 **UG-III BLAST analyses results**. Table showing BLASTN and BLASTX results of UG-III dataset with corresponding details of GB ID numbers, descriptions and E-value. Click here for file

Additional file 4 **UG-IV BLAST analyses results**. Table showing BLASTN and BLASTX results of UG-IV dataset with corresponding details of GB ID numbers, descriptions and E-value. Click here for file

Additional file 5 **UG-I Functional categorization results**. Table showing functional categorization results of UG-I dataset according to Gene Ontology accessions available at the UniProt database. Click here for file

Additional file 6 **UG-II Functional categorization results**. Table showing functional categorization results of UG-II dataset according to Gene Ontology accessions available at the UniProt database. Click here for file

Additional file 7 **UG-III Functional categorization results**. Table showing functional categorization results of UG-III dataset according to Gene Ontology accessions available at the UniProt database. Click here for file

Additional file 8 **UG-IV Functional categorization results**. Table showing functional categorization results of UG-IV dataset according to Gene Ontology accessions available at the UniProt database. Click here for file

Additional file 9 **Hierarchial clustering of UG-III contigs**. The data matrix of 105 contigs represented in hierarchical clustering dendrogram with corresponding number of ESTs represented in each library. Click here for file

Additional file 10 **List of newly developed chickpea EST-SSR primers**. The data provides information about SSR primers including details of SSR motif type, forward and reverse sequence information, melting temperature (Tm) and expected product size (bp). Click here for file

Additional file 11 **List of chickpea accessions used for screening 77 EST-SSR markers**. Chickpea genotypes used for screening newly developed EST-SSRs, with corresponding details of species, geographical origin, etc. Click here for file
